# Gemcitabine alone or in combination with cisplatin in patients with biliary tract cancer: a comparative multicentre study in Japan

**DOI:** 10.1038/sj.bjc.6605779

**Published:** 2010-07-13

**Authors:** T Okusaka, K Nakachi, A Fukutomi, N Mizuno, S Ohkawa, A Funakoshi, M Nagino, S Kondo, S Nagaoka, J Funai, M Koshiji, Y Nambu, J Furuse, M Miyazaki, Y Nimura

**Affiliations:** 1 Hepatobiliary and Pancreatic Oncology Division, National Cancer Center Hospital, 5-1-1 Tsukiji, Chuo-ku, Tokyo 104-0045, Japan; 2 Division of Hepatobiliary and Pancreatic Oncology, National Cancer Center Hospital East, Kashiwa, Japan; 3 Division of Gastrointestinal Oncology, Shizuoka Cancer Center, Shizuoka, Japan; 4 Department of Gastroenterology, Aichi Cancer Center Hospital, Nagoya, Japan; 5 Division of Hepatobiliary and Pancreatic Medical Oncology, Kanagawa Cancer Center, Yokohama, Japan; 6 Division of Gastroenterology, Kyushu Cancer Center, Fukuoka, Japan; 7 Division of Surgical Oncology, Nagoya University Graduate School of Medicine, Nagoya, Japan; 8 Department of Surgical Oncology, Hokkaido University Graduate School of Medicine, Sapporo, Japan; 9 Eli Lilly Japan K.K., Kobe, Japan; 10 Department of Internal Medicine, Medical Oncology, Kyorin University School of Medicine, Tokyo, Japan; 11 Department of General Surgery, Chiba University Graduate School of Medicine, Chiba, Japan; 12 Aichi Cancer Center, Nagoya, Japan

**Keywords:** combination chemotherapy, gemcitabine, cisplatin, biliary tract cancer

## Abstract

**Background:**

A British randomised study of gemcitabine plus cisplatin (GC) combination showed promising results in biliary tract cancer (BTC) patients. In our study, we evaluated the efficacy and safety of this combination compared with gemcitabine alone (G) in Japanese BTC patients.

**Methods:**

Overall, 84 advanced BTC patients were randomised to either cisplatin 25 mg m^−2^ plus gemcitabine 1000 mg m^−2^ on days 1, 8 of a 21-day cycle (GC-arm), or single-agent gemcitabine 1000 mg m^−2^ on days 1, 8 and 15 of a 28-day cycle (G-arm). Treatments were repeated for at least 12 weeks until disease progression or unacceptable toxicity occurred, up to a maximum of 48 weeks.

**Results:**

A total of 83 patients were included in the analysis. For the GC and G-arms, respectively, the 1-year survival rate was 39.0 *vs* 31.0%, median survival time 11.2 *vs* 7.7 months, median progression-free survival time 5.8 *vs* 3.7 months and overall response rate 19.5 *vs* 11.9%. The most common grade 3 or 4 toxicities (GC-arm/G-arm) were neutropenia (56.1%/38.1%), thrombocytopenia (39.0%/7.1%), leukopenia (29.3%/19.0%), haemoglobin decrease (36.6%/16.7%) and *γ*-GTP increase (29.3%/35.7%).

**Conclusions:**

Gemcitabine plus cisplatin combination therapy was found to be effective and well tolerated, suggesting that it could also be a standard regimen for Japanese patients.

Although biliary tract cancer (BTC) is a rare type of cancer throughout the world, it is more prevalent in East Asia and Latin America than in other countries (Matsuda and Marugame, 2007; Randi *et al*, 2009). According to ‘Demographic Statistics in Japan (2009)’ (compiled by the Statistics and Information Department, Minister's Secretariat, Ministry of Health, Labour, and Welfare (MHLW)), the number of deaths due to BTC was 17 311 in 2007, making this cancer the sixth leading cause of cancer death in Japan.

Despite great progress in diagnostic imaging, most cases of BTC are diagnosed as advanced and inoperable. Even if the tumour is not locally advanced, the primary tumour site is often contiguous with vital organs such as the liver, pancreas, or duodenum, or with major vessels such as the portal vein or hepatic artery. This anatomical peculiarity precludes resection of tumours in many cases. Furthermore, even if curative-intent surgical resection is performed, the cancer often relapses due to its invasive nature and its anatomical characteristics.

Systemic chemotherapy is usually indicated for patients with unresectable, advanced BTC or for those who have relapsed after operation; however, no standard treatment has yet been established for such patients. Gemcitabine hydrochloride is a deoxycytidine derivative that inhibits DNA elongation through intracellular phosphorylation of ribonucleotide reductase. In Japan, a single-arm Phase II study in patients with unresectable BTC confirmed that gemcitabine monotherapy had moderate efficacy and manageable toxicity, both of which were comparable with approved treatments for other cancers (Okusaka *et al*, 2006).

As gemcitabine had also been found to exhibit synergistic effects on cytotoxic activity *in vitro* and *in vivo* when combined with cisplatin (Peters *et al*, 1995; Bergman *et al*, 1996), clinical studies were conducted in various cancers with this combination. Results from these studies eventually led to use of the gemcitabine plus cisplatin (GC) combination as one of the standard treatments for non-small cell lung cancer and bladder cancer.

The combination of GC has also been studied by many researchers for the treatment of BTC (Park *et al*, 2006; Eckel and Schmid, 2007; Pasetto *et al*, 2007; Lee *et al*, 2008). So far, the largest randomised Phase III study has been the recent UK ABC-02 study, in which the efficacy and safety of gemcitabine 1000 mg m^−2^ alone *vs* the combination of gemcitabine 1000 mg m^−2^ plus cisplatin 25 mg m^−2^ was evaluated by British research groups (Cancer Research UK and University College London). That study was initiated as a randomised phase II study with gemcitabine alone *vs* GC (UK ABC-01 study) and then was expanded to a phase III study (ABC-02 study) (Valle *et al*, 2009a, 2009b).

Our study was planned to follow-up on an earlier study of gemcitabine monotherapy conducted in Japanese BTC patients (Okusaka *et al*, 2006). Given the encouraging results from the UK ABC-01 study, we conducted this study to (1) evaluate both gemcitabine monotherapy and the GC combination in Japanese BTC patients, and (2) determine whether benefits similar to those observed in the UK study could be obtained for the combination regimen.

The primary objective of the study was to compare the 1-year survival rate in patients with BTC who received one of these two therapies. The secondary objectives included response rate, progression-free survival (PFS) and assessment of safety.

## Materials and methods

### Study design

This was a multicentre, randomised phase II study to evaluate the efficacy and safety of GC combination compared with single-agent gemcitabine in chemotherapy-naive patients with locally advanced or metastatic BTC. Patients were randomised to either single-agent gemcitabine 1000 mg m^−2^ on days 1, 8 and 15 of a 28-day cycle (G-arm) or cisplatin 25 mg m^−2^ followed by gemcitabine 1000 mg m^−2^ on days 1, 8 of a 21-day cycle (GC-arm). Randomisation was stratified by primary site (gallbladder cancer or other BTC) and the presence or absence of primary tumour.

### Eligibility criteria

Eligible patients met the following criteria: histologically confirmed unresectable locally advanced or metastatic cancer of the biliary tract; no history of earlier chemotherapy; performance status of 0 or 1; a life expectancy of at least 3 months; at least 20 years of age at the time of study entry; adequate function of major organs (haemoglobin ⩾10 g per 100 ml, white blood cells ⩾3000/mm^3^, neutrophils ⩾1500/mm^3^, platelets ⩾100 000/mm3, AST/ALT/ALP ⩽3 times upper limit of normal (ULN), total bilirubin ⩽2 times ULN, ⩽3 times ULN for patients with obstructive jaundice or metastases to the liver, serum creatinine ⩽1.5 times ULN, creatinine clearance or 24-h creatinine clearance ⩾45 ml min^−1^).

This study followed the ethical principles that have their origins in the Declaration of Helsinki, and was conducted in accordance with the protocol, the ‘ordinance on Good Clinical Practice’ and related regulations. Written informed consent was obtained from all patients who were considered eligible for participation in this study before enrolment. The Efficacy and Safety Evaluation Committee, an independent review board, was consulted if any efficacy and safety issues arose in the study.

### Study treatment

The assigned treatment was given for a minimum of 12 weeks (at least four cycles in the GC-arm and three cycles in the G-arm) and continued to a maximum of 48 weeks (up to 16 cycles in the GC-arm and up to 12 cycles in the G-arm), unless disease progression (PD) was evident, an intolerable adverse event occurred or the patient was required to withdraw from the study.

### Efficacy and safety assessment

All patients who received at least 1 dose of the study drug were included in the efficacy and safety assessment. Response rate was evaluated according to the Response Evaluation Criteria in Solid Tumors. Evaluation of tumours after patient randomisation was performed every 6 weeks until PD. Adverse events were graded according to the Common Terminology Criteria for Adverse Events, version 3.0 (CTCAE v3.0).

### Statistical design and analysis

The sample size was calculated by the selection method of Simon (Simon *et al*, 1985), which is based on the proposition that GC combination therapy is selected if the 1-year survival rate for the GC-arm is higher than that for the gemcitabine arm. We assumed a 1-year survival rate of 25% for the G-arm and 35% for GC-arm (Okusaka *et al*, 2006; Park *et al*, 2006). With these assumptions, 30 patients per arm were needed to appropriately select the combination therapy with a probability of ⩾80%. To optimise safety and efficacy information, the sample size was set to 42 patients per arm.

The Kaplan–Meier method was used to estimate 1-year survival (primary outcome), PFS and 6-month PFS rates (secondary outcomes) for each treatment arm; 95% confidence intervals (CIs) were calculated. A Cox proportional hazards model was used to calculate the hazard ratio, 95% CI and its two-tailed *P*-value. Fisher's exact test was used to compare the patient characteristics, response and disease control rates, and toxicities between the two treatment arms. The exact CIs were calculated based on binomial distributions.

## Results

### Patients

**Figure 1:**
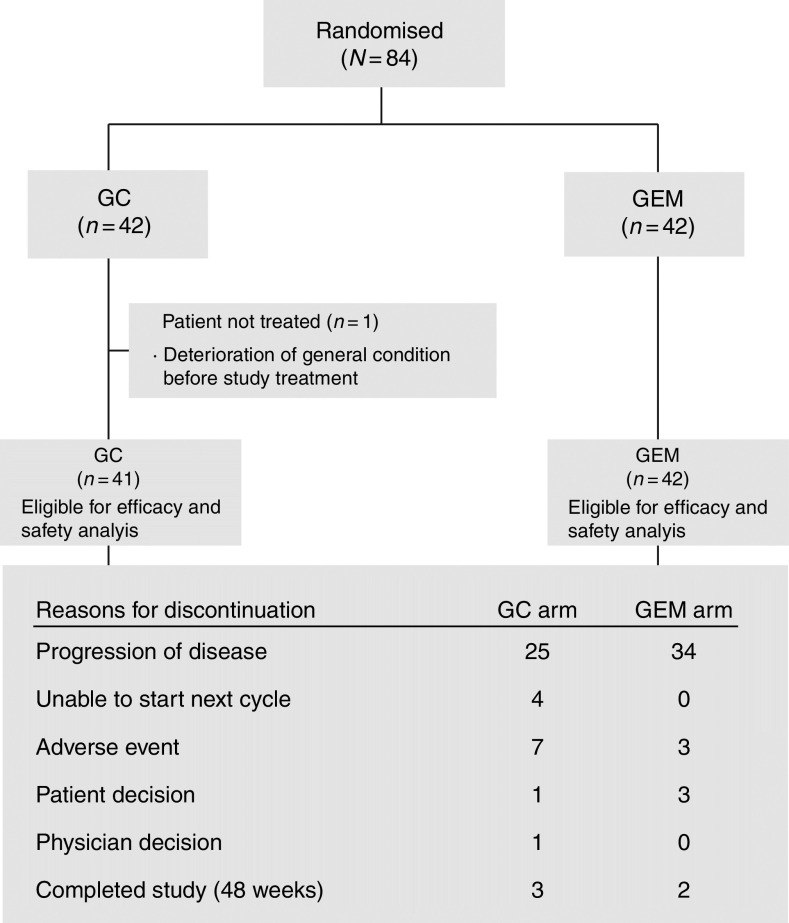
CONSORT diagram. Disposition of patients. GC=gemcitabine–cisplatin combination; GEM=gemcitabine alone.

This study was carried out from September 2006 to October 2008 at nine study centres in Japan. Eighty-four patients were randomised to either gemcitabine monotherapy (G-arm) or GC combination (GC-arm). One patient assigned to the GC-arm was not treated because the general condition of the patient deteriorated before study treatment. All of the remaining 83 patients, 41 in the GC-arm and 42 in the G-arm, received at least 1 dose of study treatment. Efficacy and safety were evaluated for each of these 83 patients (Figure 1). Demographic variables (Table 1) were well balanced between the two treatment arms, except for patients with ampullary carcinoma (4 in GC-arm, 0 in G-arm).

### Drug exposure and duration of the treatments

A total of 247 (median 6.0) and 203 (median 4.0) cycles were administered in the GC-arm and G-arm, respectively. Relative dose intensities were 78.9% for gemcitabine and 79.0% for cisplatin in the GC-arm, and 87.4% for gemcitabine in the G-arm. Three patients in the GC-arm and two patients in the G-arm completed 48 weeks treatment.

### Efficacy

**Figure 2:**
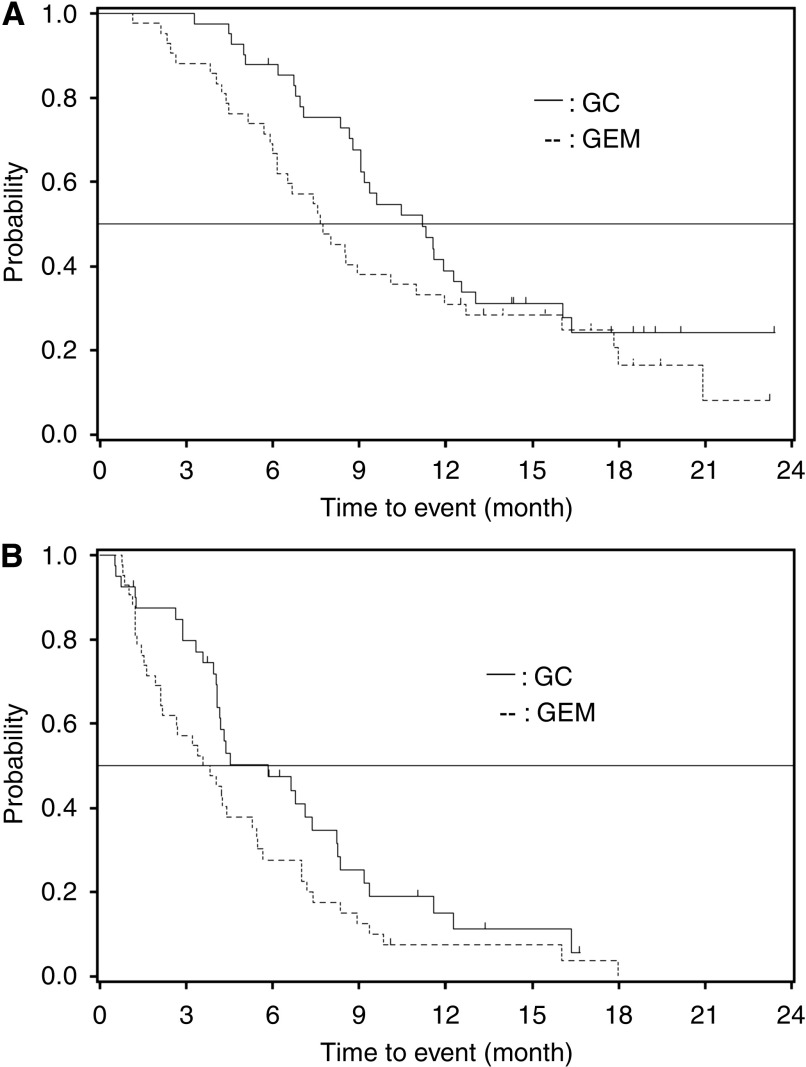
Kaplan–Meier curve of overall survival and progression-free survival. (**A**) Overall survival. (**B**) Progression-free survival. GC=gemcitabine–cisplatin combination; GEM=gemcitabine alone; CI=confidence interval.

A total of 83 patients were evaluable for tumour response according to the protocol, 41 in the GC-arm and 42 in the G-arm. No complete tumour responses were observed. In total, eight patients in the GC-arm had a partial response (PR) compared with five patients in the G-arm (PR 19.5 *vs* 11.9%). In addition, 20 patients had stable disease in the GC-arm *vs* 16 patients in the G-arm (SD 48.8 *vs* 38.1%). The disease control rate (CR+PR+SD) was 68.3% (95% CI: 51.9, 81.9) *vs* 50.0% (95% CI: 34.2, 65.8) in favour of the combination therapy. The 1-year survival rate (39.0 *vs* 31.0%), median survival time (11.2 months *vs* 7.7 months) and median PFS (5.8 months *vs* 3.7 months) were better for the GC-arm *vs* G-arm (Figure 2). The hazard ratio between the GC and G-arms was 0.69 (95% CI: 0.42, 1.13) for overall survival (OS) and 0.66 (95% CI: 0.41, 1.05) for PFS (Table 2).

As shown in Table 3, the prognosis for patients with gallbladder cancer was worse than that for patients with non-gallbladder cancer; however, the median survival times were longer with the GC combination in gallbladder cancer patients (9.1 months *vs* 6.7 months), as well as in patients with non-gallbladder cancer (13.0 months *vs* 8.0 months). The prognosis for patients with primary tumours was worse than that for patients without primary tumours; however, the GC therapy showed longer median survival time in both patient subgroups (9.4 months *vs* 7.4 months in the patients with primary tumours, 16.1 months *vs* 12.7 months in the patients without primary tumours).

### Safety

All adverse events observed in this study were predictable and manageable based on the safety profile of GC. As shown in Table 4, the most common grade 3 or higher adverse events (⩾25%) were neutropenia (56.1%), thrombocytopenia (39.0%), haemoglobin decrease (36.6%), RBC decrease (34.1%), leukopenia (29.3%) and *γ*-GTP increase (29.3%) in the GC-arm, and neutropenia (38.1%) and *γ*-GTP increase (35.7%) in the G-arm. The incidence of haematotoxicity was higher in the GC-arm; grade 3 or more serious C-reactive protein increase was detected only in the monotherapy arm.

There were no treatment related deaths. Most of the patients recovered from the above adverse events by reducing or discontinuing the study treatment.

### Post-study chemotherapy

Thirty patients in the GC-arm received post-study chemotherapy including S-1, tegaful/gimeracil/oteracil potassium (19 patients), gemcitabine (10 patients) and tegaful/uracil (1 patient). In the G-arm, 33 patients received post-study chemotherapy including S-1 (20 patients), gemcitabine (11 patients), cisplatin/fluorouracil (1 patient) and doxorubicin/tegaful/uracil (1 patient).

## Discussion

Although this study (BT22 study) showed that gemcitabine monotherapy and the GC combination were both active in Japanese patients with advanced BTC, a superior benefit was obtained with the combination treatment. In the GC/G-arms, the 1-year survival rate was 39.0%/31.0%, median survival time was 11.2/7.7 months and median PFS time was 5.8/3.7 months (Table 2).

The UK ABC-02 study, which was conducted with the same dose and regimen as this study (Valle *et al*, 2009b), showed a similar benefit for the GC combination. The respective median survival/PFS times in that study were 11.7/8.5 months in their GC-arm, and 8.2/6.5 months in their G-arm.

The hazard ratios reported in the ABC-02 study for OS (0.68, 95% CI: 0.53, 0.86) and PFS (0.70, 95% CI: 0.56, 0.88) compared well with the respective values from our study: 0.69 (95% CI: 0.42, 1.13) and 0.66 (95% CI: 0.41, 1.05). As the number of patients was based on Simon's selection method (Simon *et al*, 1985), this study was not designed to compare and identify statistical significant differences between the two treatment arms. These hazard ratios strongly suggest that the GC combination has superior benefit compared with single-agent gemcitabine, even though there were no statistical significant differences in survival and PFS between the two arms in our study.

Although there have been many single-arm Phase II studies of the GC combination for BTC (Thongprasert *et al*, 2005; Kim *et al*, 2006; Charoentum *et al*, 2007; Meyerhardt *et al*, 2008; Valle *et al*, 2009a), these results have never been distilled to one fixed dose and regimen of GC. Many previous studies of GC combination reported relatively higher response rates, but with more serious treatment-related adverse events (Thongprasert *et al*, 2005; Kim *et al*, 2006; Charoentum *et al*, 2007; Meyerhardt *et al*, 2008). In the phase II study conducted by Thongprasert *et al* (2005), 17.85% of the patients who were treated with the GC combination required dose reduction, and in another Phase II study recently conducted by Meyerhardt *et al* (2008), dose reductions and study withdrawals were required for 50% of the patients who received the combination therapy. In our study, we also observed more frequent adverse events with the doublet (Table 4). However, as shown in Figure 1, only seven patients (17%) discontinued from the study because of adverse events and four patients (9.7%) required dose adjustments in the GC-arm.

Overall, the toxicity observed in this study was manageable. Although interstitial pneumonia was detected in one patient from each of the arms, both patients recovered with appropriate treatment. One grade 3 renal failure and one grade 2 peripheral neuropathy were observed in GC-arm, in line with similar events seen in previous studies of the GC combination (Thongprasert *et al*, 2005; Kim *et al*, 2006; Charoentum *et al*, 2007; Meyerhardt *et al*, 2008; Valle *et al*, 2009a). It is to be noted that despite the higher incidence of haematotoxicity in patients receiving the combination therapy, drug-caused myelosuppression did not result in febrile neutropenia or bleeding. Grade 3 or greater increases in C-reactive protein were observed only in the gemcitabine monotherapy-arm, also suggesting that the combination therapy did not increase neutropenic infections.

In this study, we stratified patients into those with gallbladder cancer and those with other BTCs. Gallbladder cancer has been reported to have a different biological behaviour (Kim *et al*, 2006; Doval *et al*, 2004; Jarnagin *et al*, 2006); furthermore, a pooled analysis by Eckel and Schmid (2007) revealed a higher response rate to chemotherapy and shorter OS for gallbladder cancer compared with other BTCs. As shown in Table 3, patients with gallbladder cancer showed worse survival than patients with other BTCs, this being consistent with previous reports (Eckel and Schmid, 2007; Wagner *et al*, 2009). It is important to note that median survival times were longer with the GC combination in patients with gallbladder cancer (9.1 months *vs* 6.7 months), as well as in patients with non-gallbladder cancer (13.0 months *vs* 8.0 months), suggesting that the combination therapy has greater benefit than monotherapy in gallbladder cancer and other BTC patients.

Another stratification factor used for this study was the presence or absence of a primary tumour, not a commonly used stratification factor in clinical trials for advanced BTC. Locally advanced or metastatic cancer, the stratification factor used in the UK ABC-01 and UK ABC-02 studies, is more commonly used, as both of these have been shown to affect OS in advanced BTC (Park *et al*, 2009). However, considering the importance of surgical resection of the primary tumour, we decided to use this as a stratification factor for patients in this study. As shown in Table 3, patients with primary tumours showed remarkably worse survival than patients without primary tumours. However, because of the limited number of patients in our subanalyses, the results should be viewed with caution, and the usefulness of this prognostic factor should be evaluated in future studies. We will continue our efforts in collaboration with the UK ABC-02 study group to identify prognostic factors in a larger population, which may significantly affect clinical studies in BTC.

Despite the heterogeneous nature of BTC and the ethnic differences reported for this tumour type (Goodman and Yamamoto, 2007; Aljiffry *et al*, 2009), the outcomes from this study showed striking similarity with the large-scale phase III study (UK ABC-02) results. This suggests that cisplatin 25 mg m^−2^ plus gemcitabine 1000 mg m^−2^ on days 1 and 8 of a 21-day cycle would be beneficial in the treatment of advanced BTC.

## Figures and Tables

**Table 1:** Patient characteristics

	**GC (*N*=41)**	**GEM (*N*=42)**	
**Characteristic**	***n* (%)**	***n* (%)**	***P*-value**
*Gender*			
Male	18 (43.9)	21 (50.0)	0.662
Female	23 (56.1)	21 (50.0)	
			
*Age (year)*			
Median	65.0	66.5	0.0812^a^
Range	43–80	49–78	
			
*PS*			
0	34 (82.9)	28 (66.7)	0.129
1	7 (17.1)	14 (33.3)	
			
*Primary tumour sites*			
Extraheptic bile duct	8 (19.5)	11 (26.2)	0.239
Intraheptic bile duct	14 (34.1)	14 (33.3)	
Gallbladder	15 (36.6)	17 (40.5)	
Ampulla	4 (9.8)	0 (0.0)	
			
*Metastatic sites*			
Liver	22 (53.7)	20 (47.6)	0.663
Regional lymph nodes	23 (56.1)	28 (66.7)	0.372
Distant lymph nodes	19 (46.3)	18 (42.9)	0.827
Lung	8 (19.5)	7 (16.7)	0.782
Peritoneum	7 (17.1)	7 (16.7)	1.000
Bone	0 (0.0)	1 (2.4)	1.000
Others	3 (7.3)	3 (7.1)	1.000
			
*Initial onset or recurrence*			
Initial onset	30 (73.2)	32 (76.2)	0.804
Recurrence after surgery	11 (26.8)	10 (23.8)	
			
*Histological type*			
Adenocarcinoma	39 (95.1)	41 (97.6)	0.616
Adenosquamous cancer	2 (4.9)	1 (2.4)	
			
*Disease stage (gallbladder cancer, extrahepatic bile duct cancer, ampulla cancer)*			
IIA	0 (0.0)	0 (0.0)	1.000
IIB	3 (7.3)^b^	2 (4.8)^b^	
III	2 (4.9)	2 (4.8)	
IV	16 (39.0)	17 (40.5)	
Recurrence after surgery	6 (14.6)	7 (16.7)	
			
*Disease stage (intrahepatic bile duct cancer)*			
II	0 (0.0)	1 (2.4)^b^	0.389
IIIA	0 (0.0)	1 (2.4)	
IIIB	0 (0.0)	0 (0.0)	
IIIC	0 (0.0)	2 (4.8)	
IV	9 (22.0)	7 (16.7)	
Recurrence after surgery	5 (12.2)	3 (7.1)	
			
*Biliary drainage*			
No	25 (61.0)	24 (57.1)	0.824
Yes	16 (39.0)	18 (42.9)	
			
*Previous therapy*			
No	30 (73.2)	28 (66.7)	0.855
Surgery	11 (26.8)	12 (28.6)	
Radiotherapy	0 (0.0)	1 (2.4)	
Surgery and radiotherapy	0 (0.0)	1 (2.4)	

Abbreviations: GC=gemcitabine and cisplatin; GEM=gemcitabine; PS=performance status.

a *t*-test.

b Patients were diagnosed as having unresectable disease with marked regional node metastases involving the proper hepatic artery and/or main portal vein.

**Table 2:** Summary of time-to-event end points: overall response and survival

	**GC (*N*=41)**	**GEM (*N*=42)**	
	***n* (%)**	***n* (%)**	***P*-value**
*Overall response rate*			
Complete response (CR)	0 (0.0)	0 (0.0)	
Partial response (PR)	8 (19.5)	5 (11.9)	
Stable disease (SD)	20 (48.8)	16 (38.1)	
Progressive disease (PD)	9 (22.0)	17 (40.5)	
Not evaluable (NE)	4 (9.8)	4 (9.5)	
Response rate (95% CI)	19.5% (8.8, 34.9)	11.9% (4.0, 25.6)	0.380
Disease control rate (CR+PR+SD) (95% CI)	68.3% (51.9, 81.9)	50.0% (34.2, 65.8)	0.119
			
*Overall survival*			
1-year survival rate (95% CI)	39.0% (23.7, 54.4)	31.0% (17.0, 44.9)	
Median survival time (95% CI)	11.2 months (9.1, 12.5)	7.7 months (6.1, 11.0)	
Hazard ratio (95% CI)	0.69 (95% CI: 0.42, 1.13)		0.139
			
*Progression-free survival (PFS)*			
Median PFS (95% CI)	5.8 months (4.1, 8.2)	3.7 months (2.1, 5.3)	
Hazard ratio (95% CI)	0.66 (95%CI: 0.41, 1.05)		0.077
6-Months PFS rate (95% CI)	47.4% (31.4, 63.4)	27.7% (14.0, 41.5)	

Abbreviations: GC=gemcitabine and cisplatin; GEM=gemcitabine; CI=confidence interval.

**Table 3:** Overall survival time by stratification factor

**Median survival time (months)**			
**(95% CI)**	**GC (*N*=41)**	**GEM (*N*=42)**	***P*-value**
*Tumour site*			
Gallbladder	9.1 (6.9, 11.6)	6.7 (4.2, 11.0)	0.675
Non-gallbladder	13.0 (9.2, ^***^)	8.0 (6.1, 16.0)	0.110
			
*Primary tumour*			
Presence of primary tumour	9.4 (8.7, 11.6)	7.4 (5.9, 8.5)	0.253
Absence of primary tumour	16.1 (12.3, ^***^)	12.7 (6.5, ^***^)	0.389

Abbreviations: GC=gemcitabine and cisplatin; GEM=gemcitabine; CI=confidence interval.

^***^denotes upper limits are not available.

**Table 4:** Summary of maximum toxicity grades^a^ (incidence ⩾30%)

	**GC (*N*=41)**			**GEM (*N*=42)**			
	**Maximum toxicity grade**			**Maximum toxicity grade**			
**Events**	**Grade 3 (%)**	**Grade 4 (%)**	**All grades (%)**	**Grade 3 (%)**	**Grade 4 (%)**	**All grades (%)**	***P*-value**
*Haematological*							
WBC count decreased	29.3	0	87.8	19.0	0	69.0	0.061
Haemoglobin decreased	26.8	9.8	85.4	9.5	7.1	85.7	1.000
Neutrophil count decreased	39.0	17.1	82.9	28.6	9.5	69.0	0.200
Platelet count decreased	26.8	12.2	80.5	4.8	2.4	76.2	0.791
RBC decreased	34.1	0	75.6	14.3	0	78.6	0.798
Haematocrit decreased	4.9	0	58.5	0	0	54.8	0.826
							
*Non-haematological*							
Anorexia	0	0	80.5	4.8	0	61.9	0.090
Nausea	0	0	68.3	0	0	42.9	0.027
Fatigue	0	0	58.5	2.4	0	50.0	0.511
AST increased	17.1	0	53.7	14.3	2.4	52.4	1.000
ALT increased	24.4	0	51.2	16.7	0	52.4	1.000
Vomiting	0	0	48.8	0	0	23.8	0.023
GGT increased	29.3	0	46.3	31.0	4.8	50.0	0.827
Pyrexia	0	0	43.9	4.8	0	57.1	0.190
LDH increased	0	0	36.6	0	0	35.7	1.000
Constipation	0	0	36.6	0	0	33.3	0.820
ALP increased	7.3	0	31.7	16.7	0	40.5	0.495
Weight decreased	0	0	31.7	0	0	31.0	1.000
Diarrhoea	2.4	0	31.7	0	0	26.2	0.634
Blood sodium decreased	17.1	0	31.7	9.5	0	19.0	0.214
C-reactive protein increased	0	0	26.8	7.1	0	52.4	0.025

Abbreviations: ALP=alkaline phosphatase; ALT=alanine aminotransferase; AST=aspartate aminotransferase; GC=gemcitabine and cisplatin; GEM=gemcitabine; GGT=*γ*-glutamyltransferase; LDH=lactate dehydrogenase; RBC=red blood cell; WBC=white blood cell.

a Events were graded according to CTCAE v3.0.

## References

[bib1] Aljiffry M, Walsh MJ, Molinari M (2009) Advances in diagnosis, treatment and palliation of cholangiocarcinoma: 1990-2009. World J Gastroenterol 15: 4240–4262 19750567 10.3748/wjg.15.4240 PMC2744180

[bib2] Bergman AM, Ruiz van Haperen VW, Veerman G, Kuiper CM, Peters GJ (1996) Synergistic interaction between cisplatin and gemcitabine *in vitro*. Clin Cancer Res 2: 521–530 9816199

[bib3] Charoentum C, Thongprasert S, Chewaskulyong B, Munprakan S (2007) Experience with gemcitabine and cisplatin in the therapy of inoperable and metastatic cholangiocarcinoma. World J Gastroenterol 13: 2852–2854 17569122 10.3748/wjg.v13.i20.2852 PMC4395638

[bib4] Doval DC, Sekhon JS, Gupta SK, Fuloria J, Shukla VK, Gupta S, Awasthy BS (2004) A phase II study of gemcitabine and cisplatin in chemotherapy-naive, unresectable gall bladder cancer. Br J Cancer 90: 1516–1520 15083178 10.1038/sj.bjc.6601736 PMC2409709

[bib5] Eckel F, Schmid RM (2007) Chemotherapy in advanced biliary tract carcinoma: a pooled analysis of clinical trials. Br J Cancer 96: 896–902 17325704 10.1038/sj.bjc.6603648 PMC2360111

[bib6] Goodman MT, Yamamoto J (2007) Descriptive study of gallbladder, extrahepatic bile duct, and ampullary cancers in the United States, 1997–2002. Cancer Causes Control 18: 415–422 17264972 10.1007/s10552-006-0109-4

[bib7] Jarnagin WR, Klimstra DS, Hezel M, Gonen M, Fong Y, Roggin K, Cymes K, DeMatteo RP, D’Angelica M, Blumgart LH, Singh B (2006) Differential cell cycle-regulatory protein expression in biliary tract adenocarcinoma: correlation with anatomic site, pathologic variables, and clinical outcome. J Clin Oncol 24: 1152–1160 16505435 10.1200/JCO.2005.04.6631

[bib8] Kim ST, Park JO, Lee J, Lee KT, Lee JK, Choi SH, Heo JS, Park YS, Kang WK, Park K (2006) A Phase II study of gemcitabine and cisplatin in advanced biliary tract cancer. Cancer 106: 1339–1346 16475213 10.1002/cncr.21741

[bib9] Lee J, Kim TY, Lee MA, Ahn MJ, Kim HK, Lim HY, Lee NS, Park BJ, Kim JS (2008) Phase II trial of gemcitabine combined with cisplatin in patients with inoperable biliary tract carcinomas. Cancer Chemother Pharmacol 61: 47–52 17364190 10.1007/s00280-007-0444-5

[bib10] Matsuda T, Marugame T (2007) International comparisons of cumulative risk of gallbladder cancer and other biliary tract cancer, from Cancer Incidence in Five Continents Vol. VIII Jpn J Clin Oncol 37: 74–75 17272323 10.1093/jjco/hyl158

[bib11] Meyerhardt JA, Zhu AX, Stuart K, Ryan DP, Blaszkowsky L, Lehman N, Earle CC, Kulke MH, Bhargava P, Fuchs CS (2008) Phase-II study of gemcitabine and cisplatin in patients with metastatic biliary and gallbladder cancer. Dig Dis Sci 53: 564–570 17597402 10.1007/s10620-007-9885-2

[bib12] Okusaka T, Ishii H, Funakoshi A, Yamao K, Ohkawa S, Saito S, Saito H, Tsuyuguchi T (2006) Phase II study of single-agent gemcitabine in patients with advanced biliary tract cancer. Cancer Chemother Pharmacol 57: 647–653 16142487 10.1007/s00280-005-0095-3

[bib13] Park BK, Kim YJ, Park JY, Bang S, Park SW, Chung JB, Kim KS, Choi JS, Lee WJ, Song SY (2006) Phase II study of gemcitabine and cisplatin in advanced biliary tract cancer. J Gastroenterol Hepatol 21: 999–1003 16724985 10.1111/j.1440-1746.2006.04230.x

[bib14] Park I, Lee JL, Ryu MH, Kim TW, Sook Lee S, Hyun Park D, Soo Lee S, Wan Seo D, Koo Lee S, Kim MH (2009) Prognostic factors and predictive model in patients with advanced biliary tract adenocarcinoma receiving first-line palliative chemotherapy. Cancer 115: 4148–4155 19536892 10.1002/cncr.24472

[bib15] Pasetto LM, D’Andrea MR, Falci C, Monfardini S (2007) Gemcitabine in advanced biliary tract cancers. Crit Rev in Oncol Hematol 61: 230–242 17157524 10.1016/j.critrevonc.2006.04.006

[bib16] Peters GJ, Bergman AM, Ruiz van Haperen VW, Veerman G, Kuiper CM, Braakhuis BJ (1995) Interaction between cisplatin and gemcitabine *in vitro* and *in vivo*. Semin Oncol 22(4 Suppl 11): 72–79 7481849

[bib17] Randi G, Malvezzi M, Levi F, Ferlay J, Negri E, Franceschi S, La Vecchia C (2009) Epidemiology of biliary tract cancers: an update. Ann Oncol 20: 146–159 18667395 10.1093/annonc/mdn533

[bib18] Simon R, Wittes RE, Ellenberg SS (1985) Randomized phase II clinical trials. Cancer Treat Rep 69: 1375–1381 4075313

[bib19] Thongprasert S, Napapan S, Charoentum C, Moonprakan S (2005) Phase II study of gemcitabine and cisplatin as first-line chemotherapy in inoperable biliary tract carcinoma. Ann Oncol 16: 279–281 15668284 10.1093/annonc/mdi046

[bib20] Valle JW, Wasan H, Johnson P, Jones E, Dixon L, Swindell R, Baka S, Maraveyas A, Corrie P, Falk S, Gollins S, Lofts F, Evans L, Meyer T, Anthoney A, Iveson T, Highley M, Osborne R, Bridgewater J (2009a) Gemcitabine alone or in combination with cisplatin in patients with advanced or metastatic cholangiocarcinomas or other biliary tract tumours: a multicentre randomised phase II study - The UK ABC-01 Study. Br J Cancer 101: 621–627 19672264 10.1038/sj.bjc.6605211 PMC2736816

[bib21] Valle JW, Wasan HS, Palmer DD, Cunningham D, Anthoney DA, Maraveyas A, Hughes SK, Roughton M, Bridgewater JA (2009b) Gemcitabine with or without cisplatin in patients with advanced or metastatic biliary tract cancer (ABC): Results of a multicenter, randomized phase III trial (the UK ABC-02 trial). J Clin Oncol 27: 15s (Suppl; abstract 4503)(Recently the results of this study were published as follows: Valle J, Wasan H, Palmer DH, Cunningham D, Anthoney A, Maraveyas A, Madhusudan S, Iveson T, Hughes S, Pereira SP, Roughton M, Bridgewater J; ABC-02 Trial Investigators (2010) Cisplatin plus gemcitabine versus gemcitabine for biliary tract cancer. N Engl J Med362: 1273–1281)

[bib22] Wagner AD, Buechner-Steudel P, Moehler M, Schmalenberg H, Behrens R, Fahlke J, Wein A, Behl S, Kuss O, Kleber G, Fleig WE (2009) Gemcitabine, oxaliplatin and 5-FU in advanced bile duct and gallbladder carcinoma: two parallel, multicentre phase-II trials. Br J Cancer 101: 1846–1852 19904267 10.1038/sj.bjc.6605377 PMC2788250

